# Application of Agri-Food By-Products in Cheesemaking

**DOI:** 10.3390/antiox12030660

**Published:** 2023-03-07

**Authors:** Graziana Difonzo, Claudia Antonino, Giacomo Squeo, Francesco Caponio, Michele Faccia

**Affiliations:** Department of Soil, Plant and Food Sciences, University of Bari Aldo Moro, 70126 Bari, Italy

**Keywords:** cheese, by-products, antioxidant, antimicrobial, shelf life, health-promoting

## Abstract

Agri-food companies produce large quantities of plant by-products that in many instances contain functional bioactive compounds. This review summarizes the main applications of agro-industrial by-products in cheesemaking, considering their bioactivities and functional properties. Polyphenol-rich by-products increase antioxidant and antimicrobial activity in cheeses, positively impacting their shelf life. Contrasting results have been obtained regarding the color and sensory properties of enriched cheeses depending on the selected by-products and on the technology adopted for the extract preparation. Furthermore, functional compounds in cheeses perform a prebiotic function and their bioavailability improves human health. Overall, the use of agri-food by-products in cheese formulation can offer benefits for agri-food chain sustainability and consumer health.

## 1. Introduction

The incorporation of waste and by-products into food formulations to produce functional foods could meet the needs of today’s consumers and their lifestyles, with the aim of preventing the onset of diseases such as obesity, diabetes, cardiovascular diseases, etc. [1]. In addition, longer shelf life is increasingly required by both producers and consumers. Dairy producers are highly interested in extending product shelf life to enable their products to be marketed in wider markets. From this perspective, the application of functional ingredients obtained from plant by-products in cheesemaking could represent a strategy for improving the overall quality of the products.

**Figure 1:**
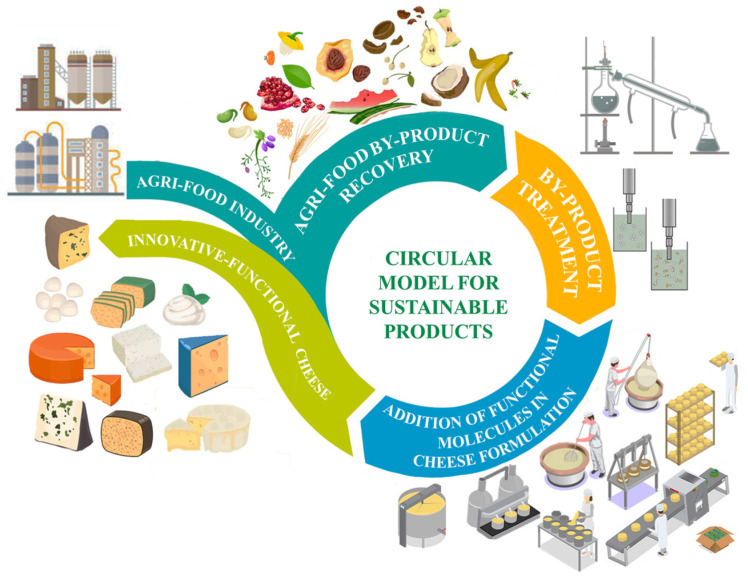
Circular model for sustainable products.

The use of agri-food by-products in food processing is well reported in the literature, based on the possibility of adding value in line with the concept of circular economy (Figure 1) [2].

Over the years, the application and efficiency of many by-products have been tested in the dairy sector. Some by-products from cucumber, citrus, pomegranate, mango, wine, and olive processing have been used to obtain extracts to be added to cheese formulations [3,4,5,6,7,8,9]. Other by-products, such as tomato peel, grape pomace, garlic and artichoke leaves, broccoli stems, chicory and cardoon roots, and cereal by-products have been applied, in the form of powders, oils, and small pieces, for the production of cow, goat, buffalo, and sheep cheeses [10,11,12,13,14]. The added by-products often had antioxidant and antimicrobial properties and exerted a positive effect on the composition, texture, sensory profile, and shelf life of the cheeses.

Cow’s milk is the milk most used for cheesemaking, with over 7,921,662 tons of cheese produced in the EU in 2022, of which Italy accounts for 13% [15]. Goat, sheep, and buffalo dairy products are produced to a lesser degree, but these types of milk are increasing in production globally and deserve to be further exploited for cheesemaking.

The aim of this review was to summarize the scientific literature on the application of various plant by-products from agri-food production chains as sources of functional compounds or as ingredients in cheesemaking. In particular, the considered studies were focused on the development of innovative cheeses by exploiting plant by-products for their technological and health-promoting properties, as well as for extending the shelf life. Furthermore, the success of cheeses enriched with agri-food by-products using less common milks was highlighted. This shows the propensity for the functionalization of less common milks, responding to the current needs of consumers who are attracted to new formulations of dairy products. This result could be useful for non-cow-milk companies, especially those present in rural and economically disadvantaged areas, as they seek to widen the typology of dairy products and find new market opportunities.

## 2. Application of Fruit and Vegetable By-Products in Cheese Formulation

The fruit industry generates millions of tons of fruit per year. In 2021, world fresh fruit production was about 907.08 million tons, and bananas, melons, apples, grapes, and oranges accounted for 50% of this total production [16]. Similarly, the production of vegetables in 2021 amounted to approximately 1154.6 million tons, with Asia and Europe being the main producers (903.19 and 89.27 million tons, respectively) [17].

Considering the large quantities of fruits and vegetables produced, it is possible that significant quantities of waste and by-products can be obtained, representing 10 to 35% of the gross mass [18]. Fruits and vegetables are ranked second in terms of food losses at 21%, preceded by roots, tubers, and oilseeds at 25%, and followed by cereals and pulses at 14% [19]. This loss can occur at processing stages including preharvest, collection, postharvest, transport, processing and packing, storage, distribution, and domestic consumption. In addition to the fruit and vegetable industries, sectors such as the winemaking industry also produce large quantities of grape pomace, grape seed, stalks, lees, and wastewater, corresponding to about 30% (*w*/*w*) of the starting quantity of grapes [20].

**Figure 2:**
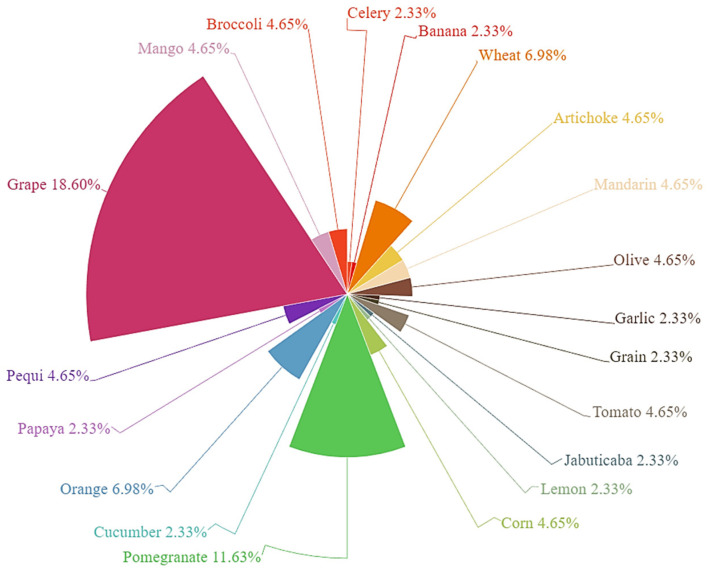
The relative percentage of the studies on the application of by-products in cheesemaking considered in this review.

Of all by-products, some are currently more widely used for the production of fortified foods, due to their higher potential. Among them, pomegranate peel and grape skin show high levels of antioxidant compounds compared to the quantities found in other by-products [21]. For this reason, their application in cheese production has been extensively tested with positive results. Other by-products from fruits, vegetables, and cereals have also been used in cheeses to verify their possible potential (Figure 2).

The potential of a by-product refers to the presence of bioactive compounds and their beneficial effects if added to food. Their quantities differ according to the type of by-product and the treatment from which it originates.

Polyphenols are among the most widespread bioactive compounds in plant by-products. They have antioxidant and radical scavenging abilities, as well as being metal chelators [22]. These characteristics provide advantages both for the quality of the products to which they are added, limiting qualitative decay, and for human health by reducing the incidence of some chronic diseases [23,24].

By-products such as roots, leaves, seeds, and skins contain dietary fiber. The intake of these compounds, which have prebiotic activity, benefits human health by reducing food-related diseases [25,26]. In food-technological terms, dietary fiber exhibits water- and oil-holding capacity, increasing viscosity and emulsification [27]. Other bioactive compounds such as vitamins, minerals, and others can be extracted from by-products for potential application in cheese formulation (Table 1).

The following chapters of this review analyze the applications of various agro-industrial by-products in cheese, summarizing the results obtained in terms of the technological characteristics of the product (shelf life, texture, sensory profile, and overall acceptability) and the nutritional features (antioxidant, prebiotic, and human health promotion). The main applications of by-products in cheese, their purpose, and results are summarized in Table 2.

## 3. Functional Properties for Cheesemaking

According to the World Health Organization (WHO) food additives “are substances added to food to maintain or improve its safety, freshness, taste, texture, or appearance”. These substances can be added to a product to fulfil a specific technological need. Each additive, before it is used in foodstuffs, must be assessed and subsequently approved to ensure that it complies with the requirements of the Codex Alimentarius and with the relevant national regulations. Therefore, by-products used as natural additives must also comply with these requirements. In addition, when using natural additives, there is the advantage of providing the consumer with a clearly labeled product that is safe and has been naturally stored.

The treatment of the by-products can influence their performance when applied in the cheese formulation. In fact, drying, freeze-drying, and microwave-vacuum-drying are some of the methods used to increase the concentrations of bioactive compounds present in plant extracts [71]. Similarly, the time and temperature of the extraction, and the type of solvent used, can increase the amounts of functional molecules in the extract obtained. Indeed, for the production of edible extracts to be added into food formulations, water and ethanol or a mixture thereof are among the best extraction solvents [72]. Thus, extraction methods and pretreatments can greatly influence the bioactive potential of an extract. Moreover, the application of different extraction methods to different matrices can lead to different results, depending on the active ingredients that the by-product contains [71]. For this reason, the potential of different agri-food by-products in cheesemaking has been discussed.

Studies on the application of by-products in cheese production have shown that the main objectives are to increase the shelf life of cheeses, thanks to the antioxidant and antimicrobial activities of functional molecules, and to study the modifications of the structure and sensory characteristics of new cheese formulations. 

### 3.1. Antioxidant Activity

Antioxidant activity refers to the inhibition of nutrient oxidation, particularly of lipids and proteins, by oxidative chain reactions [73]. This activity can be carried out by different antioxidant compounds that prolong a food’s shelf life. In cheese, the oxidation of lipids leads to the formation of hydroperoxides which can then lead to the production of off flavors. This phenomenon, followed by the release of fatty acids and the increase of microbial activity, results in qualitative decay and unacceptability of the cheese. Many antioxidant compounds such as polyphenols, tocopherols, and carotenoids are present in the by-products of fruits and vegetables, and can slow down these oxidative processes and prolong the shelf life of products [31,74,75,76]. Tripathi et al. [65] achieved an increase in the shelf life from 7 to 21 days by coating buffalo mozzarella with an edible film made from 3 mg of banana peel pectin extract. The coating resulted in an absence of yeast, mold, and coliform growth up to 21 days of storage in contrast to the uncoated mozzarella, which exhibited 2.3 × 10^2^ CFU/mg as early as the seventh day of storage. The same trend was obtained by using a gelatin–starch composite coating containing cucumber peel for fresh cow’s milk cheese. This treatment allowed the cheese to be stored for 56 days under refrigerated conditions. Furthermore, the coating provided greater product stability, limiting lipid oxidation and weight loss and thus keeping the product less hard [6].

Extracts obtained from pomegranate skin and mesocarp have been shown to produce an increase in antioxidant activity when added to the formulations of buffalo and cow cheeses [5,8,44,53,69]. These results have been obtained by applying this extract at different times and in different ways. Khalil et al. [5] added 0.25 or 0.50 g/kg of hydroalcoholic pomegranate peel extract to buffalo Feta cheese. The phenolic acids identified in high concentrations in the pomegranate peel extract were ellagic, gallic, and caffeic acids (36.24, 124, and 24.68 mg/100 g of dried extract, respectively). These resulted in higher oxidative stability of the tested cheese. In another study, Sandhya et al. [8] observed a direct proportionality between the amount of ethanolic pomegranate peel extract added to curd and the antioxidant activity found. In another study, Mahajan et al. [46] dipped Kalari cheese in an aqueous solution containing 1 and 2% of pomegranate peel extract. In this case, the antioxidant activity of the ellagic acid contained in the extract limited the lipid oxidation of cheese over 28 days of storage. In particular, the solution containing 2% of extract produced the greatest reduction of lipid oxidation. In addition, the antioxidant activity proved to be dose-dependent. Parafati et al. [53] added 0.05% (*w*/*v*) pomegranate peel and mesocarp aqueous extract to cow’s milk before coagulation. Cheese with pomegranate peel extract evidenced a higher content of total polyphenols in comparison to cheese with pomegranate mesocarp extract. The polyphenols present in higher amounts were identified as ellagic acid, punicalagin isomer, and gallagyl-hexoside (6.67, 10.24, and 8.43 mg/g of fresh vegetable material), which exhibited antioxidant activity when added to a cheese formulation in the form of peel extract. In a different study, Mushtaq et al. [69] created a biofilm with pomegranate peel aqueous extract to package Himalayan cheese. The results showed a reduction in lipid and protein oxidation in the cheeses coated with the film enriched with pomegranate peel extract. The antioxidant activity was enhanced with increasing concentration of the extract in the film. This significantly limited the oxidation of the cheese during the 30-day storage period. 

Similar results have been obtained by adding lemon and mandarin peel extract to reduce oxidation in buffalo Feta, buffalo Labneh, and processed cow’s milk cheese, respectively. The molecules with antioxidant effects when added to cheeses were ferulic acid and coumaric acid in lemon peel extract and gallic acid, catechins, coumaric acids, and rutin in mandarin peel extract, according to HPLC identification [5,7,67]. In particular, the ethanolic extract obtained from lemon peel limited oxidative damage in buffalo Feta over 28 days of storage [5]. Meanwhile, the encapsulation of aqueous extract obtained from mandarin peel was a way to preserve the antioxidative potential of bioactive compounds. In fact, the processed cheese supplemented with encapsulated extract remained unaffected after cold storage for 3 months [7].

The antioxidant activity of a by-product added to a cheese formulation depends mainly on the amounts of antioxidants present in the matrix. Their quantities vary according to the starting matrix and the antioxidant power can be influenced by intrinsic factors such as composition, geographical origin, and seasonal factors [13,14,54,60,64]; extrinsic factors such as types of treatments; and various parameters that can change the effectiveness of the by-product [77]. Frühbauerová et al. [60] highlighted how the treatment of grape peel can affect the final polyphenol content of a spreadable cheese enriched with grape peel extract. A freeze-drying treatment resulted in a higher concentration of polyphenols than drying in the oven (0.13 ± 0.02 mg GAE/100 g dry matter (DM) for control sample to 0.47 ± 0.07 and 0.54 ± 0.07 mg GAE/100 g DM for cheese with oven-dried and freeze-dried grape skin powder, respectively, identified via Folin–Ciocalteau assay). Additionally, distillation of Chardonnay grape pomace extract was shown to increase the polyphenol content in the matrix. This was confirmed by Marchiani et al. [9], who showed a higher total polyphenol content in Toma and Cheddar cheeses when enriched with distilled Chardonnay grape pomace extract compared to the same undistilled extract. The cultivar and the geographical origin also influenced the polyphenolic composition of the by-products. In particular, it was found that red grape pomace had a higher antioxidant power than white grape pomace when added to the formulations of spreadable cheese and Primosale [14,54]. Barriga-sánchez et al. [78] analyzed the antioxidant activity of Quebranta grape seeds from three different geographical areas and the results showed that seeds from zones A (167.56 ± 10.40 mg GAE/g DW and 1479.90 ± 12.86 µmol TE/g DW) and B (161.83 ± 4.95 mg GAE/g DW and 1628.15 ± 80.32 µmol TE/g DW) had higher polyphenol contents and antioxidant activities compared to those from zone C (147.63 ± 16.18 mg GAE/g DW and 1167.92 ± 106.17 µmol TE/g DW). 

For mango by-products, polyphenol contents were quantified in three different Brazilian varieties (Coquinho, Espada, and Tommy Atkins) by Posseti et al. [4]. The ethanolic kernel extracts of all three varieties showed higher polyphenol contents compared with the respective skin extracts. The kernel extract of Espada mangos showed the highest polyphenol content (15.53 mg GAE/100 g). Extracts from mango kernels were shown to be functional, improving the shelf life of Minas Frescal cheese. In another study, Khan et al. [49] showed the presence of chlorogenic acid, caffeic acid, catechin, quercetin, and mangiferin when mango kernel extract was added to Gouda cheese formulation, increasing antioxidant power.

Encapsulation is among the extrinsic factors that can increase the bioactivity of an extracts added to a cheese formulation. Farrag et al. [50] encapsulated olive mill solid waste extract to fortify white soft cheese. The addition of the extract in an encapsulated form resulted in a constant level of polyphenols in the fresh cheese and after 30 days of storage, being 125.36 and 125.95 mg/100 g, respectively. In contrast, polyphenol content decreased significantly from 112.35 to 78.95 mg/100 g cheese after 30 days of storage in the cheese fortified with free extract. The same trend was highlighted by Soliman et al. [57] for the application of wheat germ by-product encapsulation in fortified Labneh cheese. This means that the polyphenol encapsulation was a powerful strategy to keep the polyphenolic contents from degrading or oxidizing during cheese storage.

### 3.2. Antimicrobial Activity

Milk is well known as an ideal medium of microbial growth. Since the introduction of refrigeration, milk spoilage occurs mainly due to the growth of psychrophilic microorganisms that cause the breakdown of lipids and milk proteins [79]. In many cheeses, limited lipolysis and proteolysis are intended to achieve the right organoleptic characteristics, but a boost of enzymes can lead to the development of unpleasant odors [20]. These enzymes may be endogenous to the milk or result from contamination occurring during the production stages. As a result, it is important to limit the risk of microbial contamination during milking and transport, processing, and storage of these products. In addition, psychrotrophic bacteria, being thermoresistant, can remain active even after thermal stabilization processes, and special care is needed not to place the raw materials and the finished products in hazardous conditions [80].

Agri-food by-products, when used as natural additives, may prolong a product’s shelf life by limiting the deterioration caused by microorganisms. Their use has been shown to offer the possibility of limiting microbial proliferation, particularly of pathogenic microorganisms, without hindering the functional activities of starter cultures and other microorganisms useful for the cheesemaking process [12].

Pomegranate peel has been widely used for its ability to inhibit yeasts, molds, and undesired bacteria. With the aim of producing greater microbial stability and greater shelf life of the cheese, Mahajan et al. [70] added concentrations of 1 and 2% of pomegranate extract to low-fat Kalari, while Parafati et al. [53] added 0.05% (*w*/*v*) of pomegranate peel and mesocarp extract to fresh cheese. The two studies showed decreases in *Escherichia coli*, *Pseudomonas perfringens*, *Micrococcus luteus*, *Enterococci faecalis*, *Staphylococcus aureus*, *Proteus vulgaris*, *Salmonella typhii*, *Staphylococcus hemolyticus*, *Pseudomonas fluorescents*, *Bacillus subtilis*, and *Bacillus cereus*. Mushtaq et al. [69] showed that the phenolic compounds extracted from pomegranate peel have a bactericidal effect against undesirable microorganisms, allowing probiotic lactic bacteria to proliferate more easily during the preservation of buffalo Kalari cheese. This effect was also dose-dependent; increasing the extract from 25 to 75 mg resulted in an increase of diameter (mm) in the zone of inhibition of antimicrobial activity of about 5 mm for *Escherichia coli*, and 10–12 mm for *Proteus vulgaris*, *Pseudomonas perfringens*, *Microcoocus luteus*, *Enterococcus faecalis*, *Staphylococcus aureus*, and *Salmonella typhii*.

Mango peel and kernel extracts added to soft cow’s milk cheese proved to be bacteriostatic against coliform cells. The cheese with 6% extract had good microbial growth resistance during storage with limited growth of coagulase-positive *Staphylococcus* and an absence of *Salmonella* and *Listeria monocytogenes* [4].

Banana peel pectin aqueous extract used as an edible coating for fresh mozzarella cheese also showed antibacterial activity against *Escherichia coli*, *Staphylococcus aureus*, and *Salmonella enteritidis*. The limited growth of spoilage microorganisms allowed the shelf life of coated mozzarella cheeses to be increased from 7 to 21 days [66].

Pomace and olive oil by-products also showed inhibitory activity. In particular, the addition of 1% (*w*/*w*) of grape pomace powder to a fresh ovine cheese formulation favored the inhibition of *Pseudomonadaceae*, *Escherichia coli*, *Listeria monocytogenes*, *Salmonella typhimurium*, and *Stenotrophomonas maltophilia* [13]. Furthermore, the addition of this by-product did not interfere with the activity of the LAB starter cultures. Furthermore, olive oil by-products added in concentrations of 250 and 500 μg/mL retarded the growth of *Enterobacteriaceae* and *Pseudomonas fluorescens* and increased the shelf life of Fior di Latte cheese by 2 and 4 days, respectively [3].

### 3.3. Texturizing and Sensory Quality

Texturizing agents are chemicals that are added to a food to change its consistency and/or overall taste [81]. These can have a thickening, gelling, stabilizing, emulsifying, or binding role. Sensory quality is usually evaluated using human senses and is associated with a food’s appearance, smell, flavor, taste, and texture. A food may also be selected according to its sensory characteristics [82]. Texture and sensory features are strongly linked to each other; in fact, the consistency cannot be detached from the taste when a product is evaluated by the consumer. These two characteristics are then linked in the visual perception of the product [83].

Taking into account this link between texture and sensory quality in cheeses, even if a by-product is not added specifically as a texturizing or sensory enhancer, its addition will lead to a change in texture and sensory characteristics.

Lucera et al. [54] and Costa et al. [14] found that the use of wine by-products in cheese caused a change of texture. The cheeses were softer, due to a lower pH value caused by the addition of the pomace leading to a lower syneresis [14,54]; meanwhile, the hardness of Primosale cheese with added tomato, broccoli, and artichoke by-products was increased [14]. Therefore, it is important to develop products that have a formulation suitable for both functional properties and sensory acceptability. 

These obstacles have been partly overcome by the addition of extracts from by-products to cheeses in encapsulated form [7,50]. Soft cheese with an encapsulated olive by-product extract was harder than the cheese with free extract (12.40 N in cheese with free extract and 17.90 N for cheese with encapsulated extract). Gumminess and chewiness values were also higher after adding the encapsulated extract. In addition, encapsulation increased the cheese’s total solids content, protein content, and sensory acceptability [50]. However, the encapsulation process leads to the formation of additional waste from the by-products, thus generating a matrix that must be disposed of.

Indeed, in terms of sensory analysis, the addition of red and white grape pomace to spreadable and Primosale cheese allowed the identification of a marbled aspect, together with sensations of astringency, fibrousness, friability, acidity, salinity, and adhesiveness. The pomace led to greater aromatic complexity of the product. In addition, some sensations such as earthy, vinous, and taste sensations related to the addition of powder may have been limited by improved dispersion in the cheese [14,54]. The by-product can thus have less impact on sensory properties, encouraging the use of these fortifiers in cheese [13,14,54,64,84].

Cereal by-products such as wheat germ and oat bran also improved the sensory appearance of soft cow’s milk cheese [58]. Other by-products such as tomato peel and celery leaves added to a cheese formulation changed its acceptability, improving it [10,55], or, in some cases, making the product less appreciated than the corresponding conventional cheese, as was the case for mandarin peel extract added to processed cow’s milk cheese [7].

It is important to take into account the consumer acceptance of the product. In addition, informing the consumer about the addition of the by-product may influence the sensory score attributed to the latter, thereby enhancing the consumer’s appreciation of the product due to its health-promoting properties [48].

## 4. The Concept of Cheese Fortification

Food fortification or enrichment consists of adding micronutrients to food. The WHO and the United Nations Food and Agriculture Organization (FAO) define fortification as “the practice of deliberately increasing the content of an essential micronutrient in food, to improve the nutritional quality of food supply and provide a benefit to public health with the least health risk”. Enrichment, on the other hand, is defined as synonymous with fortification and refers to the addition of micronutrients to food regardless of whether the nutrients were originally present in the food before processing or not [85].

In the functional food market, fortified dairy products account for 42.9% [86]. The fortified dairy product market is constantly growing as the need for nutrient-rich foods by consumers around the world is increasing. This is positively influencing the growth of the sector, along with continuous technological advances. It is expected that the fortified dairy product market will reach almost EUR 15,675.03 million by 2027, with a compound annual growth rate of 6.9% during the forecast period [87].

From the point of view of fortification, cheese, as compared to other dairy products, is technically easier and more suitable to fortify thanks to its highly viscous nature which prevents sedimentation, a phenomenon that can happen in liquid products (beverages). This could drive demand for fortified dairy products [88].

Cheese fortification must not compromise the cheese’s physicochemical and organoleptic characteristics [61,89]. For these reasons, in fortified cheesemaking, it is important to pay attention to the type of by-product added and to its functionality, and to verify that the finished product has the characteristics that are expected. Furthermore, cheese must retain the features that make it functional. There is growing interest in using agri-food by-products as new ingredients in the dairy sector based on several studies that highlighted benefits for human health.

### Health-Promoting Applications

The perception of consumers of the importance of a balanced diet and healthy lifestyle for the prevention of diseases (cardiovascular disease, obesity, type 2 diabetes, stroke, some types of cancer, and hypertension) and for the maintenance and promotion of health is leading them to demand more functional foods [1].

A food is defined as functional when it is demonstrated that it beneficially affects one or more target functions in the body beyond adequate nutritional effects, in a way that is relevant to either an improved state of health and well-being or a reduction of risk of disease. Functional foods are different from drugs, which are aimed at preventing or curing diseases [89].

The agri-food industry over the years has modified and created new technologies to create foods that contain in their formulations functional ingredients and bioactive molecules capable of fulfilling the function of promoting human health. Fortifying cheese with bioactive compounds derived from agri-food by-products is a way to meet the growing demand from consumers [62].

Phenolic compounds are secondary metabolites produced by many plants during development or in response to stress conditions, characterized by an aromatic ring and different hydroxyl-bound groups [90]. This group of compounds includes dietary phenols such as flavonoids, phenolic acids, stilbenes, and lignans [91].

It has been demonstrated that polyphenols, due to their antioxidant capacity and capacity to regulate cellular activities, play a role in inflammatory, tumor, neurodegenerative, and cardiovascular diseases [91].

Given the richness of plant matrices in antioxidants, studies on their presence in agri-food by-products have been inevitable. The presence of polyphenolic compounds in such by-products has been widely confirmed, paving the way for their application in foods [9,14,50,60].

Numerous phenolic compounds have been identified in grape pomace and skin, such as flavan-3-ols (catechin and epicatechin), flavonols, and phenolic acids [15,60]. The addition of these winery by-products to cow’s and sheep’s milk cheeses increased antioxidant activity values and total phenols [14,54,59,60]. It has also been shown that an interaction between cheese proteins and polyphenolic by-product compounds could increase the antioxidant capacity of cheese, and that there is a positive correlation between the antioxidant activity and the total polyphenolic content of cheese [54,60].

It is important to understand how many polyphenols present in the by-products applied in cheese can have a beneficial effect on human health. It is useful to understand the levels of bioaccessibility and bioavailability of the polyphenols ingested and how these compounds interact with the molecules in the body. Polyphenols may inhibit the development of hyperlipidemia, improve glycemic control, and exert activity against glycation and glycoxidation [51]. Their ingestion can counteract the effects of consuming meals rich in sugars and lipids. Indeed, the consumption of a meal rich in these macronutrients induces oxidative stress, low-grade inflammation, and endothelial dysfunction, due to the acute increase in postprandial plasma lipids and glucose [92]. Papagianni et al. [51] highlighted that the intake of a spreadable cheese enriched with orange peel extract led to an increase in the antioxidant capacity in plasma after 3 hours in healthy subjects. The increase may have been due to a higher concentration of polyphenols in the body that was transferred to the blood plasma. In addition, in the same subjects, the increase in triglyceride levels was reduced 3 hours after the functional meal. Lowering triglyceride levels reduces the risk of cardiovascular disease, particularly arteriosclerosis. Finally, there was evidence of a reduction in the postprandial increase in glucose levels 1.5 hours after the meal with functional cheese, due to the phytochemical characteristics of the extract added to the cheese, which could inhibit carbohydrate digestion enzymes and act against glycation and glycoxidation.

## 5. Challenges and Future Perspectives on the Use of By-Products in Cheese Formulation

The application of vegetal by-products in the food industry is becoming more frequent because of the obvious benefits that can be gained from their use. However, when they are used, there are problems to be solved, both of a technological nature and related to acceptability by the consumer. The use of extracts or by-product powders in the cheese production process can lead to a change in texture that makes the product different from the standard. A future perspective could be to develop “targeted production technologies” to be used for different types of cheese that can produce the same technological characteristics as the standard product. Furthermore, many studies did not include sensory and consumer testing, which would have made the results more complete. It will therefore be useful to involve consumers in the collection of scientific results to bring better products to market in the future.

As far as the consumer is concerned, they play a very important role. They possess the purchasing power, making purchase decisions based on whether a product meets or does not meet their needs. By-products are very often seen by consumers as nonedible components or containing compounds harmful to their health. The first challenge is to inform consumers about the potential of these by-products and what their reuse in the food industry would imply both economically and environmentally. The second challenge for producers is to develop functional cheeses with good taste using fruit and vegetable by-products. Future research should focus on producing more attractive products with a better texture and flavor, as well as on marketing strategies suitable for bringing such healthy and sustainable products to the market.

## 6. Conclusions

This review highlighted the potential of numerous agri-food by-products for application in the production of functional cheeses, from both a health and a functional point of view. 

Considering the results obtained in the various studies analyzed, the agri-food by-products show strong antioxidant and antimicrobial potential. Moreover, it was possible to highlight how numerous variables modify the power of the extracts added to cheese formulations. This, therefore, confirms the possibility of carrying out further studies on different by-products, focusing above all on their final effects on cheese. This approach not only allows pursuit of the objective of improving dairy products, but above all allows the application of a circular agri-food production system with benefits for the future in terms of economic and environmental aspects.

## Figures and Tables

**Table 1:** The main agri-food by-products and their bioactive compounds.

Product	By-Product	Bioactive Compounds	Reference
Artichoke	Leaves	Dietary fibers, polyphenols, minerals	[28]
Banana	Peel	Polyphenols, pectins, biogenic amines, phytosterols	[29]
Broccoli	Stem, leaves	Nitrogen–sulphur compounds, polyphenols, vitamins, essential minerals, dietary fibers	[30]
Cardoon	Root	Dietary fibers, minerals, polyphenols, sesquiterpene lactones	[31]
Celery	Leaves	Polyphenols, vitamins, carotenoids, terpenes, coumarins, unsaturated fatty acids	[32]
Chicory	Root	Inulin, chlorogenic acids, cinnarine, lignans, quercetin	[33]
Corn	Bran, germ	Polyunsaturated fatty acids, polyphenols, tocopherols, tocotrienols, phytosterols	[34]
Cucumber	Peel	Dietary fibers, polyphenols, minerals	[35]
Garlic	Leaves	Polyphenols, vitamins, carotenoids, phytoestrogens, minerals	[36]
Grain	Brewers’ spent grain	Phenolic acids, noncellulosic polysaccharides, β-glucan	[37]
Grape	Skin, pomace, seed	Polyphenols, vitamins, stilbenes, dietary fibers	[38]
Jabuticaba	Peel	Polyphenols, dietary fibers	[39]
Lemon	Peel	Essential oils, dietary fibers, polyphenols, vitamin C	[40]
Mandarin	Peel	Polyphenols, carotenoids, vitamins, dietary fibers	[40]
Mango	Peel, kernel	PUFAs, polyphenols, carotenoids, minerals, vitamins	[41]
Oat	Bran	Peptides, amino acids, β-glucans, dietary fibers, polyunsaturated fatty acids, vitamins, minerals, polyphenols	[42]
Olive	Water, solid waste	Polyphenols, tocopherols, sterols, minerals, dietary fiber, β-carotene	[43]
Orange	Peel	Essential oils, dietary fibers, carotenoids, polyphenols, vitamin C	[40]
Pequi	Epicarp, mesocarp	Polyphenols, terpenes, flavonoids	[44]
Pomegranate	Peel	Polyphenols, flavonoids, tannins	[45]
Tomato	Peel	Lycopene, β-carotene	[46]
Wheat	Germ	Vitamins, minerals, polyphenols, polyunsaturated fatty acids, carotenoids, sterols	[47]

**Table 2:** Application of fruit and vegetable by-products in different cheeses.

Milk	Food Industry	Cheese Type	By-Product	Type	Function	Study and Key Finding	Reference
Cow	Fruit	Fresh UF cheese	Cucumber peel	Extract (polyphenols)	Shelf life improvement	A gelatin–starch composite coating containing cucumber peel (3%). ↑ Shelf life of cheese (56 days), and chemical stability. ↓ Total viable count, psychotropic bacteria, yeast, mold, weight loss, lipid oxidation, and hardness. No negative effect on the organoleptic features. Cucumber peel extract ↑ taste, odor, and texture parameters during storage.	[6]
		Petit-suisse	Jabuticaba peel	Extract (polyphenols, fibers)	Antioxidant, sensory	1.5, 2, 2.5, and 3% of extract added to cheese formulation. ↑ Polyphenols, antioxidant activity, sensory acceptability, and dietary fibers (1.65 g in formulation with 3%). Change in color parameters (↑ red value and chroma, ↓ yellow and lightness value).	[48]
		Processed cheese	Mandarin peel	Extract (polyphenols, flavonoids)	Antioxidant, texture, sensory	Cheese was made with 25, 50, and 100% (*v*/*v*) nanoliposomes of mandarin peel extract that replaced water. No significant increase of polyphenols in fortified cheese at 0 days but ↑ polyphenols during storage. ↓ In lightness value, ↑ in red and yellow value of color parameters. ↓ Organoleptic attributes.	[7]
		Minas cheese	Mango peel and kernel	Extract (polyphenols)	Antioxidant, antimicrobial, sensory	Addition of 6% of kernel and peel extracts from three varieties of mango to cheese formulation. Major polyphenol content in kernel extract. ↑ Antimicrobial activity for coliforms and coagulase-positive *Staphylococcus*. Good sensory acceptance.	[4]
		Gouda cheese	Mango kernel	Extract (polyphenols, flavonoids, fatty acids)	Antioxidant, sensory	Milk fat (3.5%) was partially replaced with mango fat at 5, 10, 15, and 20%. ↑ Polyphenols, flavonoids, antioxidant activity, C_18:1_, C_18:2_, and C_18:3_, sensory acceptability. ↓ Peroxide value, free fatty acid and cholesterol contents. Identification of mangiferin, caffeic acid, catechin, quercetin, and chlorogenic acid in cheese with mango fat.	[49]
		Soft cheese	Olive mill solid waste	Extract (polyphenols)	Antioxidant, texture, sensory	Freeze-dried and encapsulated extracts were added to cheese (100 mg/g). ↑ Polyphenol content and antioxidant activity (major content with encapsulation), flavor, body, texture and acceptability at sensory evaluation. Change in texture profile (↑ chewiness, gumminess and hardness in cheese with encapsulated extract).	[50]
		Mozzarella	Olive oil by-product	Wastewater extract (polyphenols)	Antioxidant, antimicrobial, sensory	Extract added by injection into the storage liquid in prepackaged mozzarella, 250 and 500 µg total polyphenols/mL. Polyphenols ↓ in storage liquid and ↑ in mozzarella during storage. ↑ Antimicrobial activity and shelf life (>2 and 4 days). ↓ Sensory acceptability with 500 µg total polyphenols/mL.	[3]
		Spread cheese	Orange peel	Extract (polyphenols)	Antioxidant, bioactivity	Addition of 6% of extract to cheese formulation. During postprandial phase, ↑ antioxidant activity, ↓ glucose and triglyceride levels at 1.5 h and 3 h.	[51,52]
		Curd	Pomegranate peel	Extract (polyphenols)	Antioxidant, sensory	Addition of 0.5, 1, and 1.5% of extract to milk. ↑ Polyphenols, antioxidant activity, chemical stability, and shelf life (>6 days). ↓ Sensory attributes at higher extract concentrations (astringency and bitterness).	[8]
		Soft cheese	Pomegranate peel and mesocarp	Extract (polyphenols)	Antioxidant, antimicrobial, texture, sensory	Addition of 0.5 mg/mL of extract to milk. ↑ pH cheese, polyphenols (major in cheese with peel extract at 0.1286 mg/g). Change in texture features (↑ in firmness). ↓ Lightness and redness, ↑ yellow and chroma values. ↓ *Staphylococcus* aureus and mesophilic bacteria growth during storage.	[53]
	Vegetable	Spread, Primosale cheese	Artichoke external leaves	Powder (polyphenols)	Antioxidant, texture, sensory	Addition of 5% (*w*/*w*) to spread cheese. Addition of 50 and 100 g/kg to Primosale. ↑ Polyphenols, tocopherols, and antioxidant activity. ↑ Acidity, bitterness, astringency, fibrousness, graininess, and adhesiveness.	[14,54]
		Spread, Primosale cheese	Broccoli stems and leaves	Powder (polyphenols, flavonoids, fibers)	Antioxidant, texture, sensory	Addition of 5% (*w*/*w*) to spread cheese. Addition of 50 and 100 g/kg to Primosale. ↑ Polyphenols, tocopherols, and antioxidant activity. ↑ Acidity, saltiness, astringency, hardness, fibrousness, adhesiveness, and graininess. ↓ Spreadability, solubility, and juiciness.	[14,54]
		Soft cheese	Celery leaves	Small piece (polyphenols)	Antimicrobial, texture, sensory	Addition of 5, 10, and 15% of leaves to milk used to make cheese. ↑ Antimicrobial activity. 5 and 10% ↑ sensory acceptance compared to the control sample.	[55]
		Double cream cheese	Garlic leaves	Powder (polyphenols)	Sensory, antimicrobial, shelf life	Addition of 0.8% to cheese formulation. ↑ Shelf life (>2 days), pH, dry matter, consistency, sensory acceptability, antimicrobial, and preservative activity.	[11]
		Spread cheese	Tomato peel	Lycopene oil (polyphenols)	Antioxidant, texture, sensory	Substitution of butter with lycopene oil at 0, 25, 50, 75, and 100%. Polyphenols, antioxidant activity, dry matter, ashes, texture, and sensory parameters ↑ with lycopene oil. ↓ Saturated fats with lycopene oil.	[10]
		Spread, Primosale cheese	Tomato peel	Powder (polyphenols, flavonoids, fibers)	Antioxidant, texture, sensory	Addition of 5% (*w*/*w*) to spread cheese. Addition of 50 and 100 g/kg to Primosale. ↑ Polyphenols, tocopherols, and antioxidant activity. ↑ Acidity, astringency, fibrousness, adhesiveness, hardness, and graininess. ↓ Saltiness, spreadability, solubility, and juiciness.	[14,54]
	Cereal	Processed cheese	Brewers spent grain	Powder (fibers, polyphenols)	Antimicrobial, texture, sensory	Addition of 10, 20, 30, 40, and 50% to cheese formulation. ↑ Fiber, solid, pH, meltability, texture parameters, and sensory features. ↓ Moisture, titratable acidity, proteins, and ashes.	[56]
		Spread, Primosale cheese	Corn bran	Powder (polyphenols, flavonoids, fibers)	Antioxidant, texture, sensory	Addition of 5% (*w*/*w*) to spread cheese. Addition of 50 and 100 g/kg to Primosale. ↑ Polyphenols, tocopherols, and antioxidant activity. ↑ Sweetness, astringency, fibrousness, adhesiveness, hardness, graininess. ↓ Acidity, saltiness, spreadability, solubility, and juiciness.	[14,54]
		White cheese	Wheat germ	Encapsulated oil (polyphenols)	Antioxidant, texture, sensory	Cheese with fat replaced by 50% with free or encapsulated wheat germ oil. Compared to the control, lightness ↓ with free oil, redness and yellowness ↓ with encapsulated oil and ↑ with free oil. ↑ Flavor, body and texture, and appearance. ↓ Sensory attributes with free oil, ↑ with encapsulated oil.	[57]
		Soft cheese	Wheat germ	Extract (polyphenols)	Antioxidant, sensory	Addition of 0.5 and 1% of hot and cold extract to cheese formulation. Extract ↑ moisture, pH, and proteins compared to the control sample. ↑ Sensory features during storage.	[58]
	Winery	Petit Suisse	Grape seed and skin	Seed extract, skin flour (polyphenols, flavonoids, anthocyanins)	Antioxidant, sensory	Addition of grape skin flour (3 g/100 g) and grape seed extract (0.5 g/100 g) to cheese formulation. ↑ Polyphenols, antioxidant activity, and sensory appreciation (↑ odor and appearance, ↓ taste and consistency).	[59]
		Toma, Cheddar	White and red grape pomace (Barbera, Chardonnay)	Powder (polyphenols)	Antioxidant, antimicrobial	Addition of 0.8 and 1.6% (distilled and not distilled powder) to cheese formulation. In Toma, ↓ moisture and fat (except 0.8% Chardonnay, not distilled), ↑ proteins and ashes. In Cheddar, ↑ moisture and proteins (except with Chardonnay, not distilled), ↑ ashes and ↓ fat (except 1.6% Chardonnay, not distilled). Slightly higher proteolysis with powder. Significant ↑ antioxidant activity and polyphenols with 1.6% of the powder. ↑ Organic acids, particularly lactic acid ↑ in Toma and ↓ in Cheddar. Distillation ↑ bioactive compounds in cheeses.	[9]
		Spread, Primosale cheese	White and red grape pomace	Powder (polyphenols, flavonoids, fibers)	Antioxidant, texture, sensory	Addition of 5% (*w*/*w*) to spread cheese. Addition of 50 and 100 g/kg to Primosale. ↑ Polyphenols, tocopherols, and antioxidant activity. ↑ Acidity, sweetness, astringency, fibrousness, adhesiveness, and graininess. ↓ Saltiness, sweetness, spreadability, hardness, solubility, and juiciness.	[14,54]
		Spread cheese	White grape skin	Oven-dried and freeze-dried powder (polyphenols, flavonoids)	Antioxidant	Addition of 1 and 2% (*w*/*w*) of powders to cheese formulation. With extract, ↑ proteins, dry matter, and ashes, ↓ fat. ↑ Polyphenols, flavonoids, and antioxidant activities in cheese with freeze-dried powder.	[60]
		Robiola-type	White and red skin (Barbera and Chardonnay)	Powder (polyphenols, flavonoids)	Enrichment, antioxidant, sensory	Addition of 0.8, 1.6, 2, and 4% (*w*/*w*) to curd. A major percentage ↓ sensory attributes and consumer appreciation.	[61]
Goat	Fruit	Minas Frescal cheese	Pequi epicarps and external mesocarps	Extract (polyphenols)	Antimicrobial, texture	Addition of extract to milk and mass, and cheese immersion in the extract (6.25 mL/L). ↑ Titratable acidity, redness and yellowness, hardness. ↓ Lightness and cohesiveness. Pequi extract addition to milk was the most stable in terms of bacteriological parameters. The extract delay postacidification contributed to preservation of texture and color.	[12,62]
Sheep	Fruit	Ricotta cheese	Orange peel	Powder (polyphenols, β-carotene)	Antioxidant, antibacterial	Addition of 3% (*w*/*v*) of powder in edible coating formulation to preserve the cheese. ↑ Antimicrobial and antioxidant activities, lightness, and shelf life. ↓ Redness and textural parameters.	[63]
		Primosale cheese, Vestedda-like	Red grape pomace	Powder (polyphenols, flavonoids)	Antioxidant, antimicrobial, texture, sensory	Addition of 1% (*v*/*v*) in curd. ↑ Proteins, moisture, dry matter, redness index, volatile profile, sensory parameters, and antioxidant and antimicrobial activities. ↓ Fat, saturated fatty acids, and lightness and yellowness.	[13,64]
	Cereal	Feta	Wheat bran	Powder (polyphenols)	Antimicrobial, sensory	Addition of 10% of wheat bran to cheese formulation. ↑ Prebiotic growth, volatile compounds, sensory features, and shelf life.	[65]
Buffalo	Fruit	Mozzarella	Banana peel	Pectin extract (polyphenols, flavonoids)	Antioxidant, antimicrobial	Edible coating with 3 mg of pectin. ↑ Antioxidant and antimicrobial activities, and shelf life from 7 to 21 days. Absence of yeast, mold, and coliform growth up to 21 days of storage in contrast to the uncoated mozzarella.	[66]
		Feta-like	Lemon peel	Extract (polyphenols, fatty acids)	Antioxidant, antimicrobial, sensory	Addition 0.25 and 0.5 g/kg to milk for cheesemaking. ↑ Total solids, fat, proteins, pH, volatile fatty acids, antioxidant and antimicrobial activities, and sensory properties (best 0.5%). ↓ Peroxide and acid values.	[5]
		Labneh cheese	Mandarin peel	Extract (polyphenols, fibers)	Antioxidant, antimicrobial, sensory	Addition of 1.5, 3, and 5% extract to cheese formulation. ↑ Total solid, proteins, ashes, carbohydrates, acidity, fibers, antioxidant and antimicrobial activities, *Lactobacillus helveticus* growth, and sensory properties (best with 3%). ↓ pH value.	[67]
		Kareish cheese	Papaya leaves	Extract (polyphenols)	Antioxidant, antimicrobial, sensory	Addition of 2% of extract to the edible coating. ↑ Tenderness, juiciness, body, texture, and flavor. Improvement of shelf life and antimicrobial and antioxidant activities.	[68]
		Feta-like	Pomegranate peel	Extract (polyphenols, fatty acids)	Antioxidant, antimicrobial, sensory	Addition of 0.25 and 0.5 g/kg to milk for cheesemaking. ↑ Total solids, fat, proteins, pH, volatile fatty acids, antioxidant and antimicrobial activities, and sensory properties (best 0.5%). ↓ Peroxide and acid values.	[5]
		Himalayan cheese	Pomegranate peel	Extract (polyphenols)	Antioxidant, antimicrobial, sensory	Zein film with 0, 25, 50, and 75 mg/g of film. Use of biofilm to package cheese. ↑ Barrier properties, antioxidant and antimicrobial activities, sensory acceptability. ↓ Oxidation.	[69]
		Kalari cheese	Pomegranate rind	Extract (polyphenols, fatty acids)	Antioxidant, antimicrobial, sensory	Immersion of cheese in an aqueous solution of 1 and 2% extract. ↑ Moisture, antioxidant and antimicrobial activities, shelf life, and sensory scores. ↓ pH, lipid oxidation, and free fatty acids.	[70]

↓ = decrease; ↑ = increase.
